# Changes in DNA methylation during epigenetic-associated sex reversal under low temperature in *Takifugu rubripes*

**DOI:** 10.1371/journal.pone.0221641

**Published:** 2019-08-27

**Authors:** He Zhou, Zi-Xin Zhuang, Yu-Qing Sun, Qi Chen, Xin-Yi Zheng, Yu-Ting Liang, Shahid Mahboob, Qian Wang, Rui Zhang, Khalid A. Al-Ghanim, Chang-Wei Shao, Ya-Juan Li

**Affiliations:** 1 Key Laboratory of Marine Bio-resources Sustainable Utilization in Liaoning Province’s University, Dalian Ocean University, Dalian, China; 2 Key Laboratory of Mariculture, Agriculature Ministry, PRC, Dalian Ocean University, Dalian, China; 3 Department of Zoology, College of Science, King Saud University, Riyadh, Sandi Arabia; 4 Yellow Sea Fisheries Research Institute, CAFS, Key Lab for Sustainable Development of Marine Fisheries, Ministry of Agriculture, Qindao, China; Massachusetts General Hospital, UNITED STATES

## Abstract

DNA methylation has frequently been implicated in sex determination and differentiation in teleost species. In order to detect the DNA methylation patterns established during sexual differentiation in tiger pufferfish *T*. *rubripes*, we performed comprehensive whole genome methylation sequencing and analyses of the gonads of male, female, and pseudo male. We obtained a total of 33.12, 32.44, and 31.60 Gb clean data for male, female, and pseudo male, with a sequencing depth of 66.44×, 60.47× and 54.86×, respectively. The methylation level of cytosine (C) residues in the genomic DNA from gonads was 11.016%, 10.428%, and 11.083% in male, female, and pseudo male, respectively. More than 65% of C methylation was at CpG sites, and less than 1% was at CHG and CHH sites. In each regulatory element, there were low methylation levels on both sides of the transcription start site, and higher methylation levels in exons, introns, and downstream of genes. The highest mCpG was on chromosome 8 and the lowest mCpG was on chromosome 5. Comparisons of whole-genome DNA methylation between pairs of samples revealed that there were 3,173 differentially methylated regions (DMRs) between female and male, and 3,037 DMRs between male and pseudo male, corresponding to 0.232% and 0.223% of the length of the genome, respectively. There were only 1,635 DMRs between female and pseudo male, representing 0.127% of the length of the genome. A number of differentially methylated genes (DMGs) related to sex determination and differentiation were selected, such as *amhr2* and *pfcyp19a*. After Bisulfite Sequencing PCR (BSP) verification, *amhr2* was exhibited low methylation level in normal males and pseudo male, and high methylation level in normal females but *pfcyp19a* showed low methylation level in normal females and high methylation level in normal males and pseudo males. These results provide information about the molecular epigenetic mechanisms of DNA methylation during low-temperature induced masculinization of tiger pufferfish, and increase our understanding of the mechanisms of sex determination and differentiation in this important aquaculture fish species.

## Introduction

Sex determination and differentiation is a fundamental developmental process that establishes the sexual genotype at the early stage of an organism and then destines the undifferentiated gonad into ovary or a testis [1, 2]. Teleost fishes with 25,000 species are the most diverse group of vertebrates and consequently have the most complicated sex determination mechanisms ranging from genetic sex determination (GSD) to environmental sex determination (ESD) [2]. In some fish species, sex is determined by genetic factor but also largely dependent on the environmental factors (eg. temperature, salinity, density, pH and social interactions) [2, 3]. This has led to desire to produce mono sex population of interest for aquaculture by artificial manipulation with examples of all male population of tilapia (*Oreochromis mossambicus*), yellow catfish (*Pelteobagrus fulvidraco*) and all female population of Japanese flounder(*Paralichthys olivaceus*) and chinook salmon (*Oncorhynchus tshawytscha*) [4–7].

The tiger pufferfish *T*. *rubripes*, commonly known as ‘fugu’, is one of the most popular aquacultural fish in China, Korea and Japan [8]. Traditionally, the testes of tiger pufferfish were considered as a delicacy and thus the male fish have a higher economic value than females [9]. Tiger pufferfish has an XY sex determination system with environmental influence, especially the temperature during the sex determination sensitive stages [10]. Therefore, all male production by treatment of the temperature was extensively performed in tiger pufferfish. Previous studies have shown that a low-temperature treatment in the 2–3 weeks after hatching significantly increased the proportion of male tiger pufferfish [11]. Recently, Zhou et al. [12] obtained a population with 75% male tiger pufferfish by incubating fertilized eggs for 45–75 days at 20 °C, and then for 35–65 days at 15–17 °C. Meanwhile, by extensive genetic analysis, a sex determination gene (*amhr2*) residing on the chromosome 19 was identified and a single nucleotide polymorphism (SNP) in *amhr2* associated with sex determination that can be used for molecular sexing in sex reversal fish by low temperature [13]. Although the sex reversal has been successful in practical, little is known about the molecular mechanism of sex determination and differentiation under low temperature in tiger pufferfish.

Epigenetics was considered as a bridge between genetics and environment, which refers to changes in gene expression that do not involve changes in the DNA sequence [14]. DNA methylation is a major epigenetic modification and an important regulator of gene function [15]. In general, gene expression levels are negatively correlated with DNA methylation [15]. Various environmental factors, especially temperature, affect DNA methylation patterns in fish species [16–20]. Recent studies confirmed that DNA methylation play an important role in fish sex determination and differentiation. In Chinese tongue sole (*Cynoglossus semilaevis*), the high temperature (28 °C) can induce sex reversal of juvenile genotypic females into phenotypic pseudo-males by epigenetically regulation and more interestingly such epigenetic modification can be passed down to the ZW offspring of pseudo males in normal temperature [17]. In European sea bass (*Dicentrarchus labrax*), the methylation levels of the *cyp19a* promoter in female increased under high temperature, leading to inhibition of gene expression [18]. An increase of even 2 °C can lead to global changes in DNA methylation in European sea bass larvae [19]. High temperature has also been shown to increase gene methylation levels in Nile tilapia (*Oreochromis niloticus*) [20].

To date, the role of DNA methylation in the low-temperature-induced masculinization of tiger pufferfish is unclear. We inferred that low temperature treatment in tiger pufferfish may change the DNA methylation pattern and further affect gene expression levels, and finally cause significant phenotypic changes, the sex reversal. In this study, we obtained a population by low-temperature treatment containing pseudo male fish as well as female and male fish. The sex of each fish was identified by analyzing the specific SNP locus in *amhr2* and by physiological analyses. We then conducted whole genome bisulfite sequencing (WGBS) of DNA from gonads of male, female, and pseudo male tiger pufferfish, and constructed a whole-genome methylation map at single-base resolution. These analyses revealed details of the epigenetic mechanism of low-temperature induced masculinization and thus provided the theoretical basis for breeding all male population of tiger pufferfish.

## Materials and methods

### Ethics statement

This study was performed according to the Guide for the Care and Use of Laboratory Animals in Dalian Ocean University, Dalian, China. All animal experiments were approved by the Animal Study Ethical Committee of Dalian Ocean University, and comply with Chinese laws, regulations, and ethics.

### Fish sample collection

The fish samples used in this study was produced by the low-temperature treatment induced masculinization of fugu in Zhou et al. [21]. Briefly, an orthogonal test L_9_ (3^4^) design was chosen with three factors at three levels: treatment starting times (days post-hatch, dph) of 20, 50, 80 dph; treatment temperatures of 13 °C, 15 °C and 17 °C; treatment duration of 30, 45, 60 days. The control group was reared at 21±1 °C. The experiments were repeated twice. Conduct vivisection until development to 230 days of age, gather the fin structure in 95% alcohol and the gonads in cryogenic vials, preserved at -80 °C. These experiments were conducted at Daheishi Aquaculture Facility of TianZheng Industrial Co., Ltd.

### Screening of *T*. *rubripes* pseudo males

Pseudo males were screened in the following two groups: 1. 20 days after hatching, treating the offspring for 30 days at 13 °C; 2. 80 days after hatching, treating the offspring for 60 days at 13 °C.

To identify sex, we used the specific SNP gender identification markers developed by Kamiya [13]. Caudal fin tissue (30 mg) was ground in 95% alcohol, and high-quality DNA was extracted using the phenol/chloroform extraction method. The DNA concentration was determined by spectrophotometry, and then diluted to 50 ng/ml. The PCR primers used were SD3exon8F (ACGATGCACACAAACCACCT) and SD3exon10R (TCCCAGTGTTGCGGTATGTA). Each 50 μl PCR reaction mixture contained 5.0 μl Taq buffer, 1.0 μl dNTPs (10 mmol/L), 1.0 μl upstream and downstream primers, 5 μl MgCl_2_ (25 mmol/L), 0.5 μl Taq DNA polymerase (5 U/μl), 1.0 μl template DNA, and 35.5 μl double-distilled water. The PCR cycling conditions were as follows: 95 °C pre-degeneration for 3 min; then 35 cycles of 94 °C for 30 s, enaturation at 55–60 °C for 35 s, and extension at 72°C for 40–50 s; followed by final extension at 72 °C for 5–8 min. The reaction products were kept at 4 °C until further analysis.

The gender was also assessed by a tissue sectioning technique. Gonads were collected from fugu in the low-temperature treatment group and control group (30 per group) at 230 days of age. The gonads were fixed in Bouin’s solution, and then subjected to gradient alcohol dehydration before clearing and embedding in xylene paraffin. Sections (6–8 μm thick) were cut using a microtome, stained with hematoxylin-eosin, sealed in neutral rubber, and then observed and photographed under an Olympus microscope (Olympus, Tokyo, Japan).

### Construction and sequencing of genome-wide bisulfite methylC-seq libraries

We extracted DNA from gonads (frozen at -80 °C) of five individuals of male (average body length: 140.35±7.68 mm, average body weight: 84.94±6.30 g), female (141.04±4.44 mm, 85.88±6.86 g), and pseudo male (121.18±6.69 mm, 50.73±10.68 g) using the phenol/chloroform method. Equal amounts of DNA samples from male, female, and pseudo male were mixed and then broken into fragments (approximately 250 bp average size) using a Bioruptor Ultrasonic Cell Disruptor (Diagenode, Sparta, NJ, USA). The 3′ ends were repaired by adding adenine with methylation connectors. The bisulfite treatment was conducted using an EZ-DNA Methylation-Gold kit (Zymo Research, Orange, CA, USA). The fragments were separated by 2% agarose gel electrophoresis, recovered for PCR amplification using a QIAquick Gel Extraction kit (Qiagen, Hilden, Germany), and used to build the methylC-seq enrichment library. When we built the methylation library, an internal reference phage DNA was added, the conversion rate could be obtained by counting the phase DNA. We used the lllumina HiSeq2000 platform for high-throughput sequencing.

### Data analyses

We used the Solexa Pipeline -1.0 for basic data processing (e.g. filtering). After filtering, clean data was compared with the reference genome (Assembly accession: No. GCA_000180615.2) by BSMAP [22] to calculate the rate of bisulfite conversion. When read coverage ≥ 4, supported methylation read numbers > unsupported methylation read numbers, we could determine the methylation site. We then calculated the mthelation level for an individual CpG using the formula:

Rmaverage=Nmall/(Nnmall+Nmall)×100%

(where Nm is the read number of methylC, and Nnm is the read number of methylC at the locus) [23]. For the methylation level of a specific region, it was calculated by the sum of methylation levels of individual CpGs divided by the total number of covered CpGs in the region.

We next searched for the 500 bp sliding windows containing at least five CG (CHG or CHH) at the same position of the genome in two samples, and compared the difference in CG methylation levels in the window between the two samples. Regions showing a significant difference in methylation levels (2-times difference, Fisher’s test *P* value ≤ 0.05) between two samples were defined as DMRs. If a continuous region of two adjacent DMRs showed differences in methylation levels between two samples, the two DMRs were either merged as a continuous DMR or treated as two separate DMRs. We further performed the Kyoto Encyclopedia of Genes and Genomes (KEGG) enrichment analyses of the genes in the DMRs.

### Bisulfite sequencing method

The genome-wide methylation sequencing analysis of fugu revealed several genes related to gonad development. These genes were selected for further analysis by BSP. Primers were designed using Primer Premier 5.0. For the *amhr2* the primers were as follows: Forward, TTTGTTATTTATGGTGTT TTTAGATG; Reverse, CAAAAAAATATCAAAACAAAAATAAAC. For the *pfcyp19a* the primers were as followers: Forward, TAGGAGGGATGATAGAAATATGAAG; Reverse, CAAACC AACTAAATAATAAACAATACAC. High-quality DNA was extracted from the gonads by the phenol/chloroform method. To reduce variation in the male, female, and pseudo male samples, each DNA sample was a mixture of DNA extracted from gonads of three *T*. *rubripes* individuals. The EZ-DNA Methylation-Gold kit (Zymo Research) was used for genomic DNA sulfite modification. The PCR reaction conditions were as follows: 4 min at 98 °C; 20 cycles of 45s at 94 °C, 45s at 66 °C, 1min at 72 °C, 20 cycles of 45s at 94 °C, 45s at 56 °C, 1 min at 72 °C and a final extention of 8 min at 72 °C. PCR products were detected by gel electrophoresis and recycled by SanPrep Column DNA Gel Extraction Kit (Sangon). The recycled DNA was cloned into the pUCm-T Vector and transformed in *E*. *coil* compent cells (Sangon). Then 20 clones per loci for each sample were sequenced. The sequencing results were analyzed using DNAStar software.

### qPCR analysis

qPCR was used to examine the different expression quantity of *amhr2* and *pfcyp19a* gene in female, male and pseudo male gonad. Total RNA was reverse transcribed to first-strand cDNA using RevertAid Premium Reverse Transcriptas kits (#EP0733; Thermo Scientific, Waltham, MA, USA). The reactions were performed in an ABI Step One Plus machine (Thermo Scientific). Primer Premier 5.0 was used to desgined the primers for these genes. For the *amhr2* the primers were as follows: Forward, TGTGCGATTTCGGATGTTC; Reverse, CGTGCATCAGATAC CATTTGTT. For the *pfcyp19a* the primers were as follows: Forward, TACC GAAGGGCACAAACATC; Reverse, GAACCGAACGGCTGAAAGT. The cDNA samples diluted 10 times served as test templates. The reaction mix included SybrGreen qPCR Master Mix 10 μl, 0.4 μl fragment F (10 μM), 0.4 μl fragment R (10 μM), 7.2 μl ddH_2_O and 2 μl template cDNA. The following thermal cycling parameters were used: initiation at 95 °C for 3 min followed by 45 cycles of 95 °C for 7 s for denaturation, 57 °C for 10 s for annealing, and 72 °C for 15 s for extension. All experiments were conducted with three biological replicates for each sample. The relative expression levels were normalized to the endogenous control geneβ-actin, and expression ratios were calculated by using the 2^-ΔΔCt^ method. Statistical analysis among the results of female, male and pseudo male gonad was performed using Duncan’s multiple range alignment and significance tests with SPSS version 17.0 (Statistical Product and Service Solutions Inc., Chicago, IL, USA).

## Results

### Sex identification

**Fig 1:**
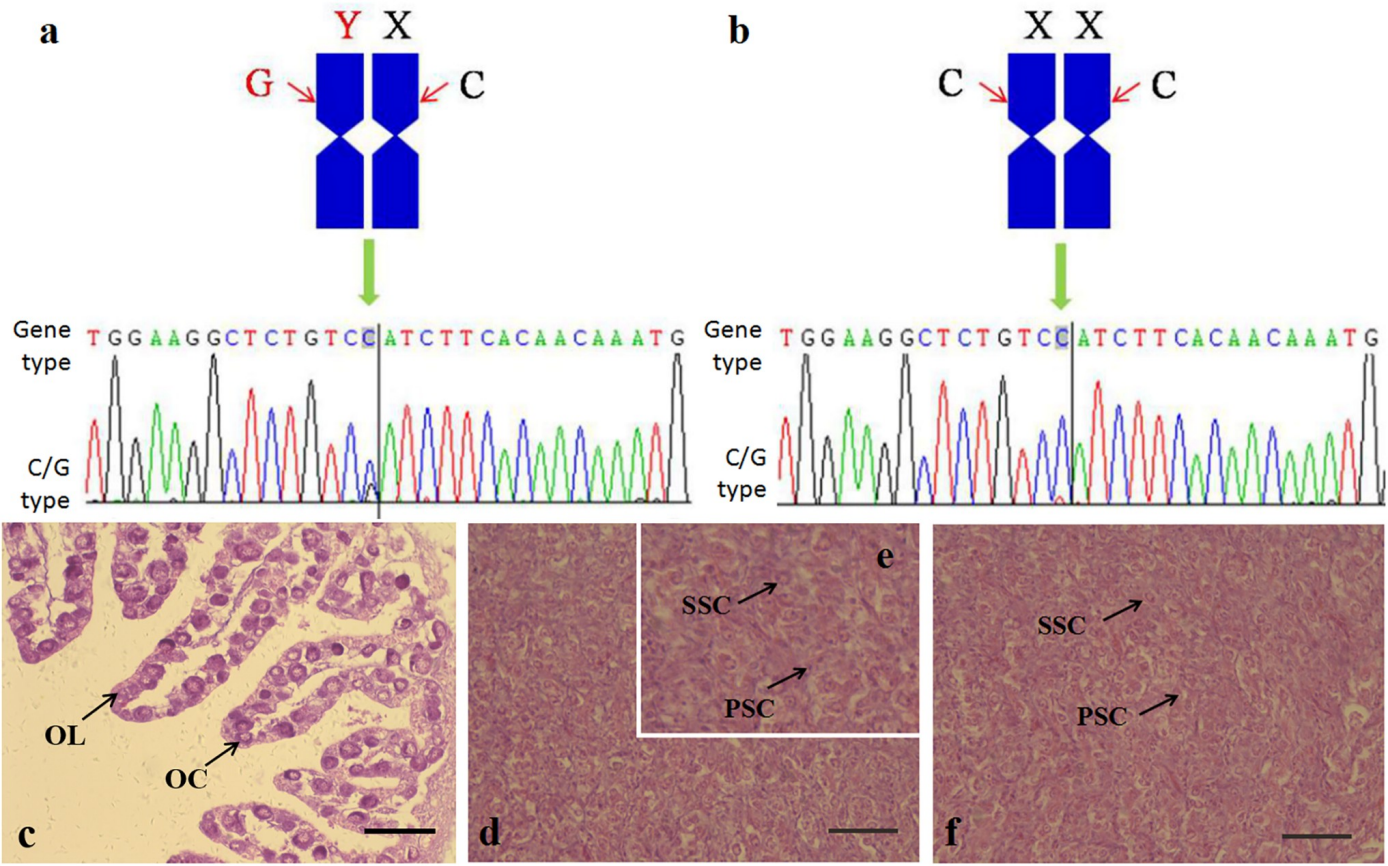
Genetic and phenotypic sex identification in *T*. *rubripes*. **a**: normal male; **b**: normal female and pseudo male; **c**: normal female ovary; **d** pseudo male testis; **e**: pseudo male testis amplification; **f**: normal male testis; **OC**: oocytes; **OL**: ovarian lamella; **PSC**: primary spermatocytes; **SSC**: secondary spermatocytes; bars = 100 μm.

The genotypic sex of tiger pufferfish was identified by SNP analysis of *amhr2* genes using PCR method. The SNP at the *amhr2* loci should be C/G in male (Fig 1a), and C/C in female and pseudo male (Fig 1b). The phenotypic sex of tiger pufferfish was identified using a histological analysis. The histological sections of gonad revealed that the testes structure was similar in male and pseudo male tiger pufferfish (Fig 1c–1f). In total, 37 male, 23 female and 5 pseudo male tiger pufferfish were identified.

### Whole genome methylation sequencing data

The DNA extracted from gonads of male, female and pseudo male was used for genome-wide methylation sequencing by illumina HiSeq2000 platform. We obtained 34.64, 33.24, and 33.83 Gb raw data for male, female, and pseudo male, respectively. After removing the low-quality data and contaminants, a total of 33.12, 32.44, and 31.60 Gb clean data for male, female, and pseudo male, with a sequencing depth of 66.44×, 60.47× and 54.86×, respectively, were generated for further analysis (Table 1). The genome coverage was higher than 95% for all samples and the mapping rate for each sample is 71.35%, 66.78% and 63.11% for male, female and pseudo male respectively. In the three different samples, the proportion of C in different forms covered by at least two reads was greater than 97% (S1 Table). The data has uploaded to NCBI Sequence Read Archive (SRA accession: PRJNA524991).

**Table 1:** Methylation sequencing data for male, female and pseudo male tiger pufferfish.

Sample	Raw data(Gb)	Clean data (Gb)	Average depth (×)	Genome Coverage (%)	Mapping rate (%)	Bisulfite conversion rate (%)
**Male**	34.64	33.12	66.44	95.04	71.35	99.60
**Female**	33.24	32.44	60.47	95.06	66.78	99.61
**Pseudo male**	33.83	31.60	54.86	95.03	63.11	99.58

### Methylation features in tiger pufferfish genome

**Fig 2:**
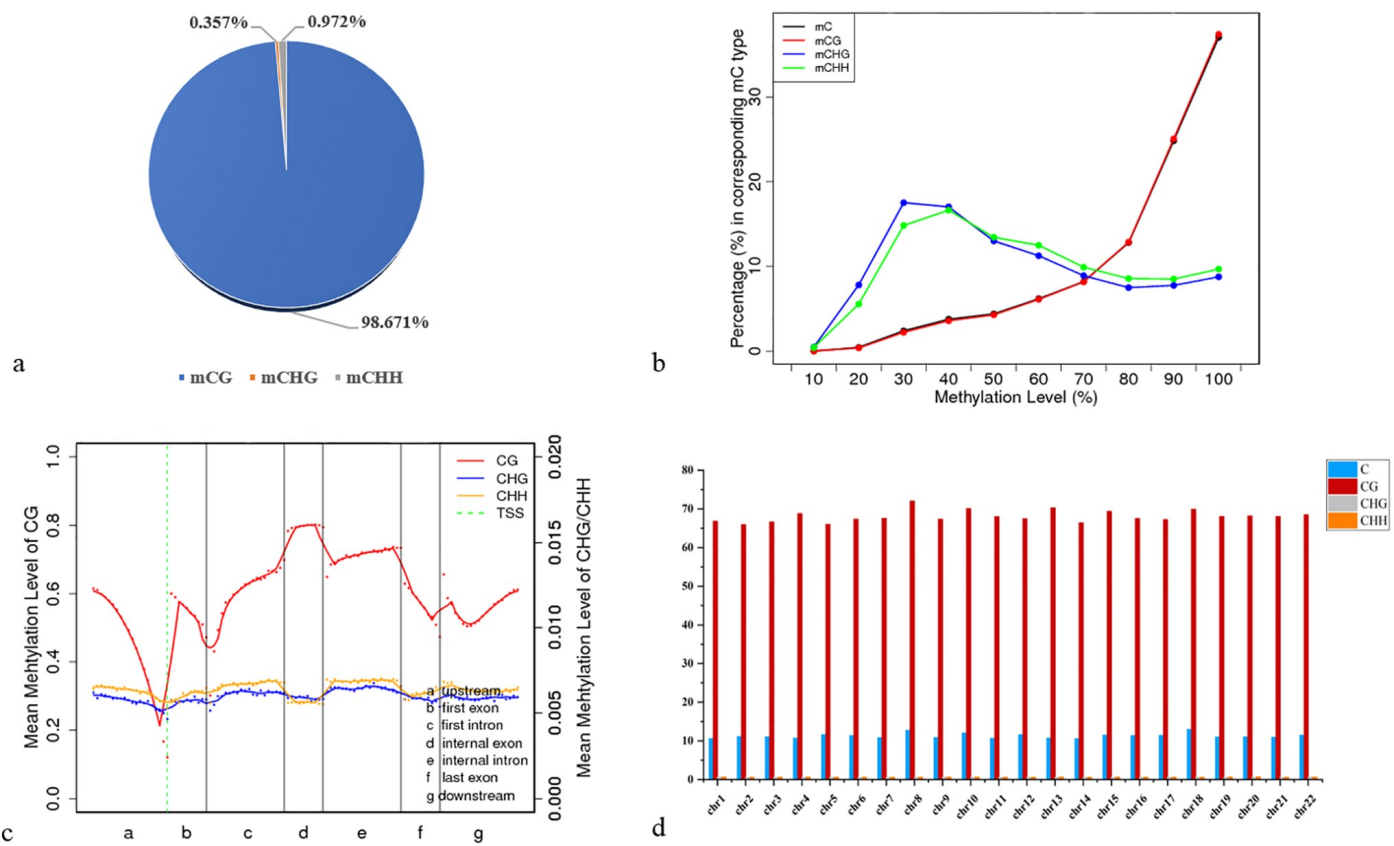
Methylation features in male tiger pufferfish genome. **a**: Distribution ratio of C methylated of different sequence types; **b**: Methylation patterns of methylated C. X axis was the methylation level, from left to right is 0% ~ 100%, 10% each for a Y axis, the proportion of specific methylation level of mC for all mC, the methylation level of C base is equal to the C base sites on the support sequence of the site for effective coverage methylation sites in the proportion of the value in the sequence; **c**: Methylation patterns of different functional element regions in the whole genome. The dotted line between a and b is the TSS position; **d**: Methylation level of each chromosome.

We identified an average of 13.2 million methylated cytosines (mCs) accounting for 11% of Cs in each sample (S2 Table). The classification of mCs showed a similar proportion in male, female and pseudo male genome. More than 98% of mCs were mCG type while about 0.3% and 0.9% were mCHG and mCHH types in the CpG context. Thus, it can be seen that genomic methylation of each gender occurred mainly at CG loci (Fig 2, S2 Table and S1 Fig). The mCpG (ratio of mC to CpG dinucleotide sites) was about 65% (66.009%, 65.027% and 65.945% for male, female and pseudo male respectively) (S2 Fig). We thus focus solely on CpG sites for subsequent analyses. We found the CG methylation level was between 60% and 70% in exons, introns, mRNA, and non-coding RNA, which is much higher than in tRNA (an average of 20.2%) (S3 Table). We further analyzed the methylation status of CpGs in various genomic elements. The lowest methylation level was detected around TSS but increased within the exons and introns with the peak at internal exon, which was exhibited the similar methylation level in male, female and pseudo male. (Fig 2 and S1 Fig). Furthermore, we investigated the DNA methylation on a chromosomal level. The mCpG is around 65% for each chromosome and the highest mCpG was on chromosome 8 (72.024%, 71.686%, and 73.471% for male, female and pseudo male, respectively) and the lowest mCpG was on chromosome 5 (65.985%, 60.006% and 60.659% for male, female and pseudo male, respectively) (Fig 2 and S3 Fig).

### Methylation density analysis

According to the chromosome level density distribution of methylation C bases, with 10 kb area as a unit length, the single-base resolution methylation pattern of female, male and pseudo male was been printed. In pseudo male, methylation density of both ends in some of the chromosomes were greater than the middle area, such as chromosome 1. And in few chromosome, methylation density of ends is greater than the other area density, such as chromosome 3 (S4 Fig). In male and female, the density distribution in the form of probability showed the similar situation with pseudo male.

### DMRs and the corresponding genes

**Fig 3:**
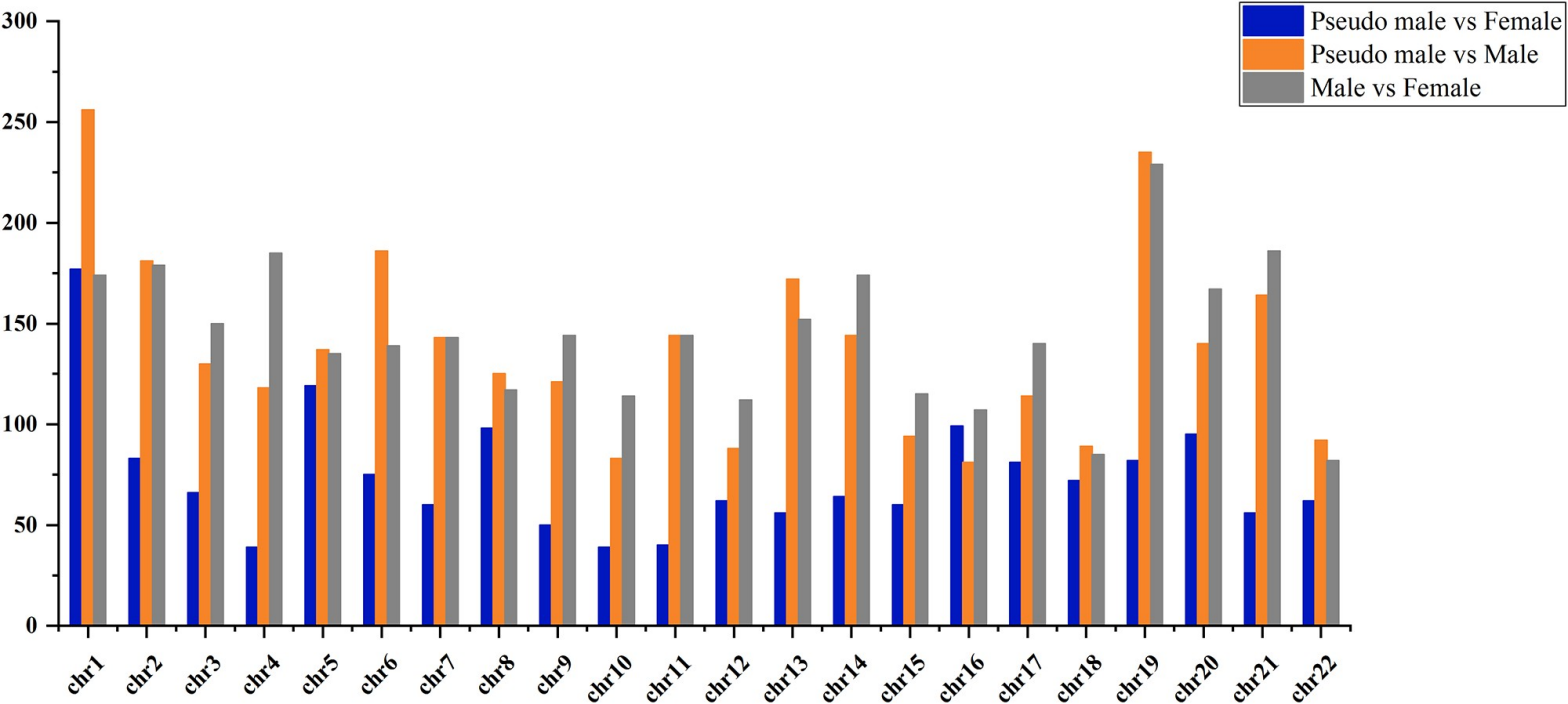
The distribution of DMRs on each chromosome in tiger pufferfish.

**Fig 4:**
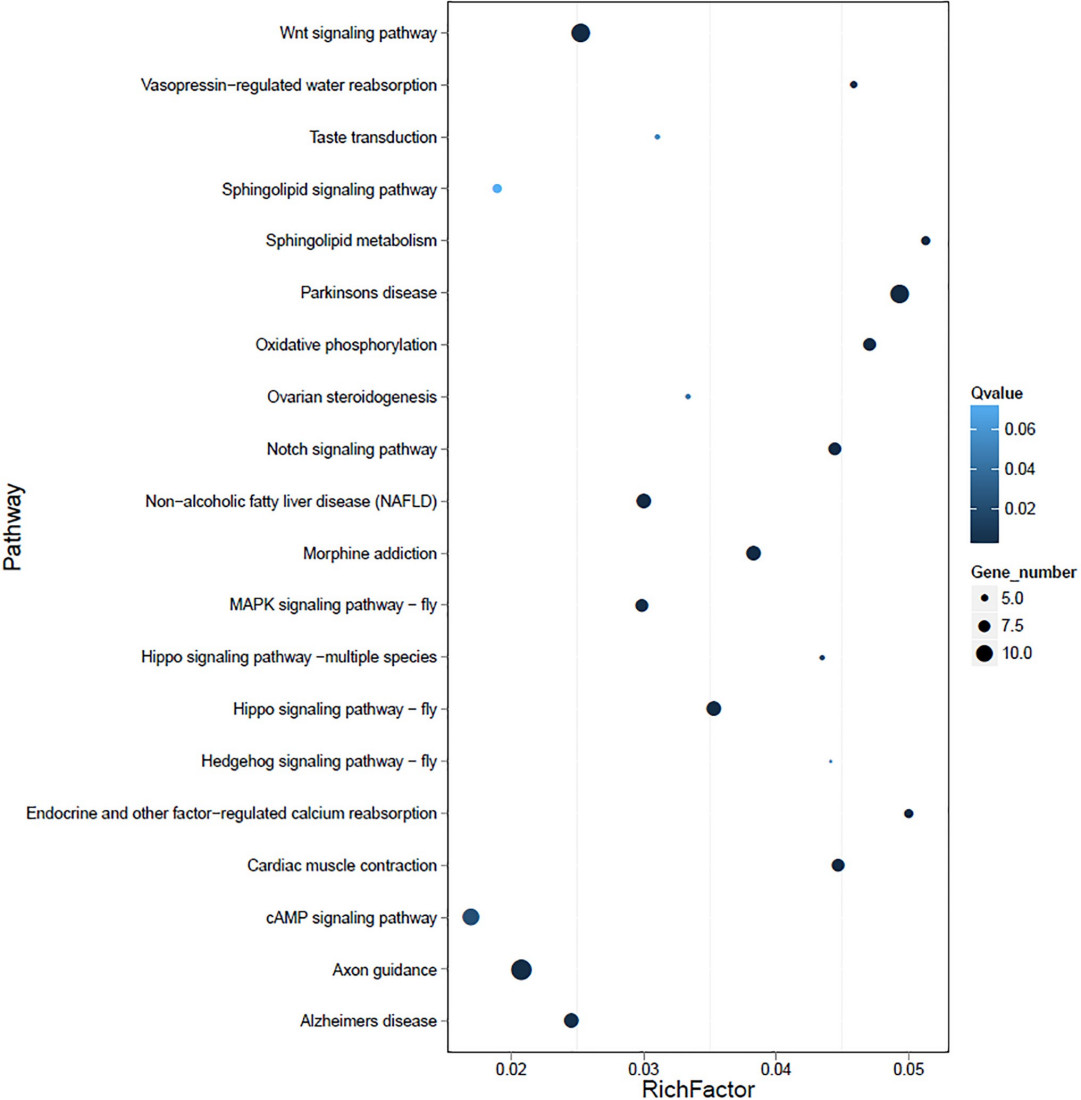
The scatter plot for KEGG enrichment of DMGs on the sex chromosome of tiger pufferfish. Rich Factor is the ratio of differentially expressed gene numbers annoted in this pathway terms to all gene numbers annoted in this pathway term. Q ≤ 0.05 as significantly enriched.

We made pair-wise comparisons of male, female, and pseudo male using a 10 kb sliding window method to detect the DMRs. In total, the 3,173 DMRs with a length of 898,849 bp covering 1,931 differentially methylated genes (DMGs) were identified between male and female. Besides, the 1,635 DMRs between pseudo male and female and 3,037 DMRs between pseudo male and male were identified with a length of 498,023 bp and 909,539 bp, representing 966 and 1,782 DMGs, respectively. Of note, the sex chromosome (Chr. 19) contained the highest number of DMRs (229) and 121 DMGs between male and female. Both the highest number of DMRs (256 between male and pseudo male and 177 between female and pseudo male respectively) were located on the chromosome 1. The distribution of DMRs on each chromosome are shown in Fig 3. Furthermore, the DMGs were functionally clustered via the KEGG. We observed a strong enrichment in different biological processes for DMGs in each pair-wise comparison and notably, all the DMGs in sex chromosome were enriched in functions associated with the embryonic or gonad development such as Notch signaling pathway, Wnt signaling pathway and Ovarian steroidogenesis (Fig 4).

### Verification of differentially methylation genes

**Fig 5:**
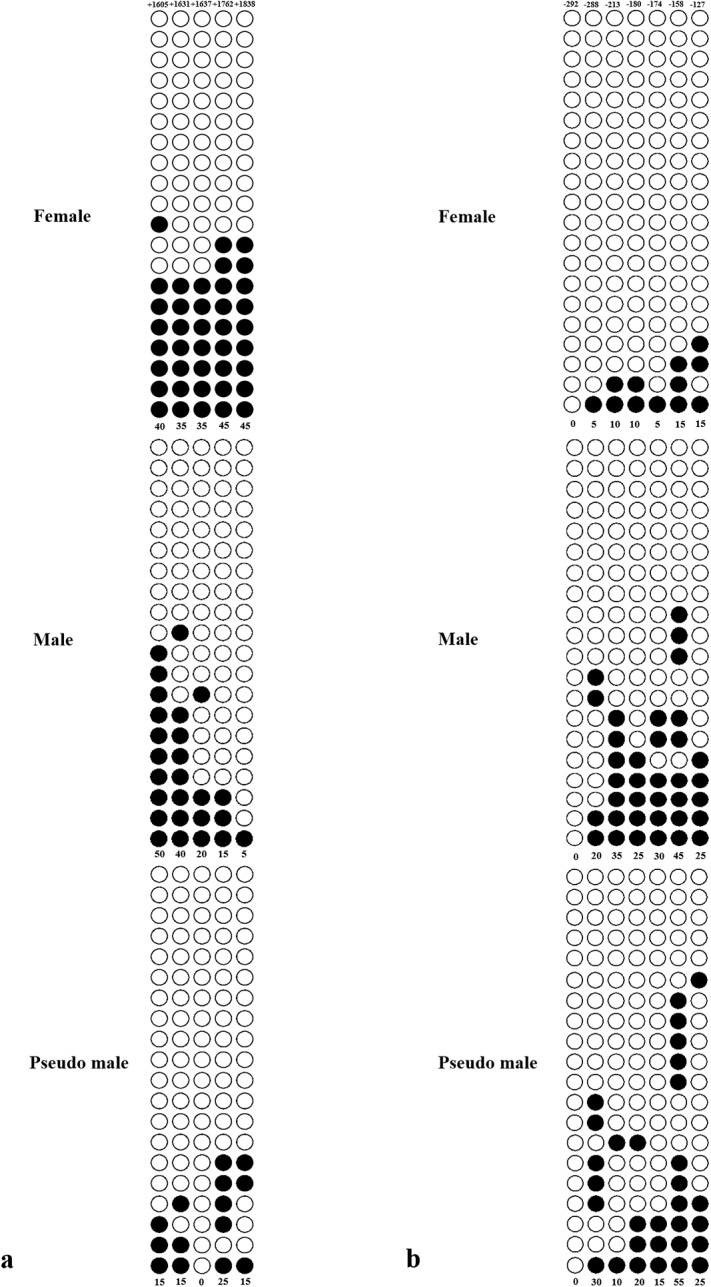
*amhr2* and *pfcyp19a* gene promoter and the first exon of CpG island methylation analysis of *T*. *rubripes*. a: *amhr2*; b: *pfcyp19a*. Solid circles represent methylated C, hollow circles represent unmethylated C, without the circle line represents the missing value (may be due to sequencing errors). Numbers with a plus or minus sign indicate CpG positions with respect to the transcription starting site.

Differential methylation genes screening several genetic variations were related to sex differentiation, such as *amhr2*, *zglp1*, *nobox*, *dmrt1*, *foxl2* and *pfcyp19a*. Two genes (*amhr2* and *pfcyp19a*) were selected for BSP verification and the results showed the difference between both sexes in the methylation level of promoter region of CpG island in the promoter and first exon. 40% (40/100) of the CpG sits in promoter and the first exon in *amhr2* gene in female ovaries were methylated, while 26% (26/100) in normal male testis and 14% (14/100) in pseudo male testis (Fig 5a). Besides, 8.6% (12/140) of the CpG sits in promoter and the first exon in normal female ovary was methylated, and 25.7% (36/140) and 22.1% (31/140) in the testis of normal males and pseudo males, respectively in *pfcyp19a* gene (Fig 5b).

### Expression of differentially methylation genes by qPCR

**Fig 6:**
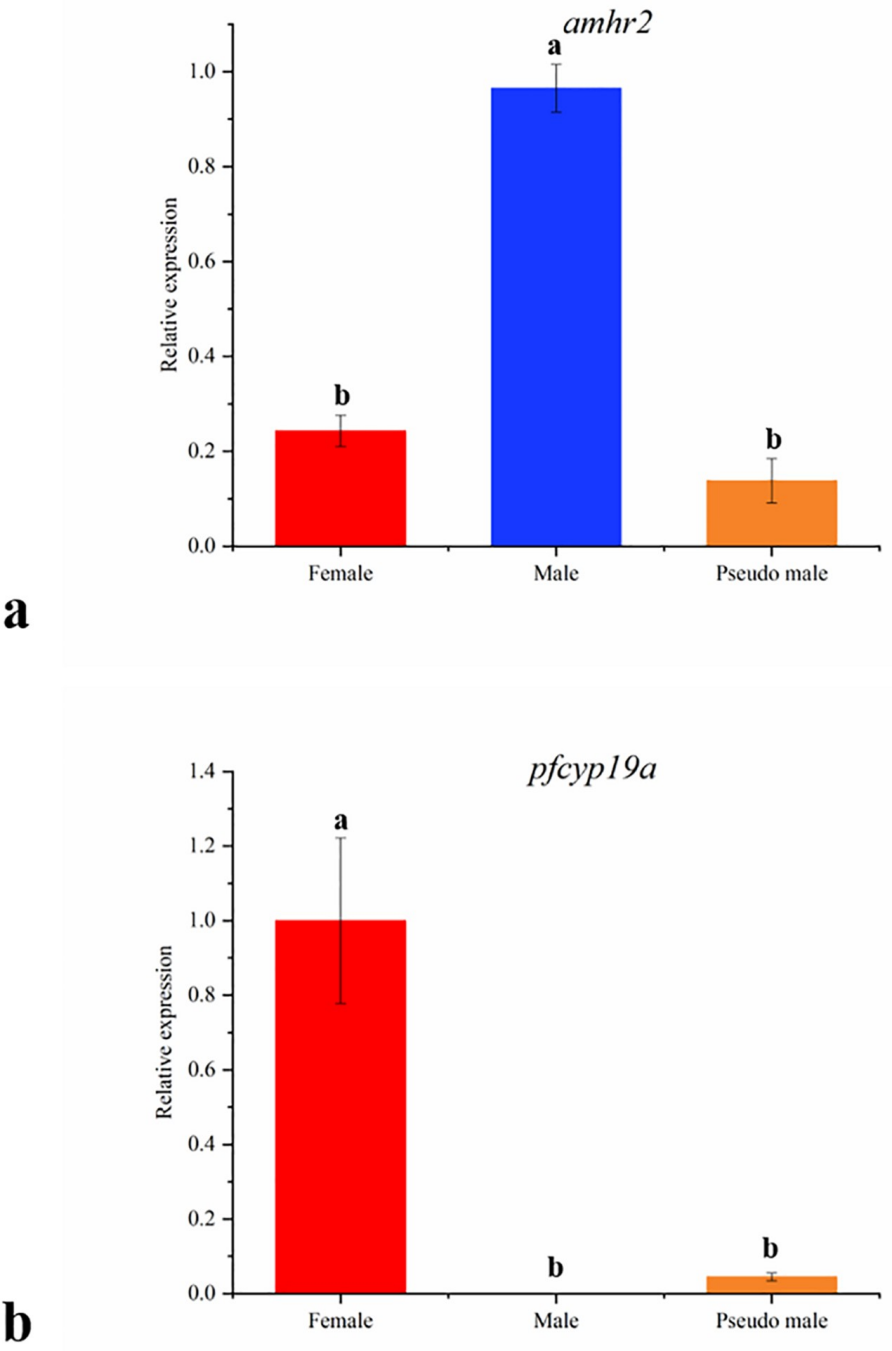
qPCR expression of *amhr2 and pfcp19a* in female, male and pseudo male gonad. a: *amhr2*; b: *pfcyp19a*. Different letters indicated significant differences (*P* < 0.05) while the same letters indicated that the differences were not significant (*P* > 0.05).

qPCR was performed on *amhr2*, *pfcyp19a* gene for detecting the expression pattern among female, male and pseudo male. For *amhr2*, the relative expression of male is higher than female and pseudo male, female is close to pseudo male (Fig 6a). For *pfcyp19a*, the relative expression of female is higher than male and pseudo male, male is close to pseudo male (Fig 6b).

## Discussion

With the rapid development of next generation sequencing technology, several techniques for detecting the genome-wide DNA methylation were developed including the WGBS [23], reduced representation bisulfite sequencing (RRBS) [24], methylated DNA binding domain sequencing (MBD-seq) [25] and methylated DNA immunoprecipitation sequencing (MeDIP-seq) [26]. Comparatively speaking, WGBS are more accurate and can detect the methylation status with an unbiased view and frequency of each CpG locus in the whole genome, making it better to determine the effect of DNA methylation on various aspects of growth, development, and immunity [27]. Our study presents the first whole-genome methylation map through WGBS in tiger pufferfish and gives a comparatively investigation of the DNA methylomes among sexual systems.

To date, there have been few reports on DNA methylation at a whole genome level of fish species. It was firstly reported that it has heavy methylation exclusively at CG sites in the *Tetraodon nigroviridis* genome [28]. Recently, the dynamics of DNA methylomes at a single-base resolution for zebrafish gametes and early embryos reaveled that the sperm DNA methylome can be inherited in zebrafish early embryos [29]. Also, comparative analysis of the kinds of gonadal DNA methylomes in Chinese tongue sole revealed that epigenetic regulation controlled temperature-related sex reversal in this fish species [17]. Another example in tilapia, a number of significant clusters of sexually dimorphic DMRs on chromosomal regions associated with sex determination were detected, suggesting the DNA methylation involving into the sex-specific phenotypes [30]. In this study, we determined the effect of temperature on the sex determination and differentiation of tiger pufferfish from an DNA methylation perspective, and revealed how environmental factors interact with the epigenetic mechanism of sex determination.

Our analysis on the male, female and pseudo male methylomes in tiger pufferfish revealed that about 11% of C is methylated in the genome, with more than 98% of C methylation at CpG context. This result is consistent with genome methylation patterns reported for above fish species. In Chinese tongue sole, about 9% of mCs in each sample while more than 99% of mCs were in the CpG context [17]. Other fish species above showed similar patterns with high mC in CpG context. This phenomenon is also observed as in other veterbrate genomes such as human [23] and chicken [31], suggesting that C methylation patterns are highly conserved in vertebrates. Besides, our analyses revealed that the methylation level was lower in controlling elements than in other gene parts. Specifically, the methylation levels were lower on both sides of TSSs, and higher in exons, introns, and downstream genes. These results are consistent with those reported for *T*. *nigroviridis* [28], Chinese tongue sole [17] and indicate that the methylation level of the promoter is negatively correlated with gene expression as reported for other vertebrates [32, 33]. This gene structure CpG methylation pattern may be explained by functional constraints within genic regions to prevent the frequency of mutations [34]. All of these observations on the distribution of DNA methylation in gene structure showed largely conserved from teleost to humans and consistent with the enriched CpG dinucleotides in functional regions (eg. promoter regions) of the genome.

DNA methylation as a mediator of environmental change is a gene expression regulatory mechanism in adaptive phenotypic variation [35]. In this study, we observed the methylation alterations in the genome level accompanying by the sex reversal of tiger pufferfish induced by low temperature. The male tiger pufferfish showed higher methylation level (66.009%) than in female (65.027%) and the pseudo male showed an increase in methylation level (65.945%) which is close to methylation level in male after low-temperature induction. This phenomenon was also observed in Chinese tongue sole and tilapia. The methylation level was consistently enhanced by 10% in pseudo male gonad compared with female gonad after high temperature induction in Chinese tongue sole [17]. Similarly, the tilapia gonad showed an increase in methylation level induced by high temperature at a genome wide [20]. Interestingly, no matter high or low temperature treatment, it can increase in methylation levels resulting in sex reversal in fish species. These results suggested that environmentally-induced DNA methylation can cause the phenotypic plasticity, the sex reversal induced by temperature for above instance, which provides a substrate for adaptive phenotypic variation triggered by epigenetic regulation.

Sexually dimorphic patterns of DNA methylation in the genomic context, particularly in sex chromosome or sex-associated regions have regularly been implicated on the sex determination and differentiation in teleost species [30]. Our analysis identified the most DMRs (3,173) between female and male and followed by 3,037 DMRs between pseudo male and male, 1,625 DMRs between pseudo male and female, which were relatively enriched in sex chromosome (229, 235, and 82). This result suggested the DMRs in sex chromosome are sensitive to environment, which play a key role in epigenetic modification on sex differentiation of tiger pufferfish. We further found enrichment in DMGs in sex chromosome in a few functionally well-characterized pathways, namely ovarian steroidogenesis. Genes associated with gonadal development were selected for BSP verification and expression analysis. Generally, the CpG island is mainly located in the promoter region and the first exon region, hypermethylation of CpG in promoter regions and enhancer regions inhibits the expression of genes [36–38]. Besides, most of sex related genes exhibited cellular-specific expression pattern during the development of gonad. *amhr2* is the master male sex-determining gene in tiger pufferfish and it was expressed in the Sertoli cells and Leydig cells in the testis [39]. *pfcyp19a* encodes aromatase P450A that converts androgens into estrogens and it was expressed in the follicular cells and granulosa cells around oocytes [40, 41]. In this study, the *amhr2* gene is hypermethylated in female but hypomethylated in male and correspondingly it shows low expression in females and high expression in males. However, the hypomethylation of this gene in pseudo male did not resulting into its high expression. These results suggest that there may be more regulatory approaches to sex-determining genes during the sex reversal of tiger pufferfish. Conversely, the patterns of negative correlation between methylation and expression in the promoter of *pfcyp19a* in pseudo male is possibly involved in a male transition in tiger pufferfish.

In conclusion, we performed comprehensive whole genome methylation sequencing and analyses on the gonads of tiger pufferfish and further observed a pattern of an increase in methylation level in pseudo male tiger pufferfish after low temperature treatment. We also identified the DMRs enriched in the sex chromosome and the corresponding pathways that involved in the gonad development in tiger pufferfish. These results not only contribute to knowledge about the sex control of fish, but also provide a scientific basis for the production of all-male tiger pufferfish population.

## Supporting information

S1 Table Effective coverage rates of different forms of C in male, female and pseudo male. (XLSX)

S2 Table Methylation pattern ratio of male, female, and pseudo male. (XLSX)

S3 Table Methylation level of all types of regulatory elements in whole genome. (XLSX)

S1 Fig The average methylation level of the whole genome of *T*. *rubripes*. (TIF)

S2 Fig Methylation features in female (a-c) and pseudo male (d-f) tiger pufferfish genome. **a, d**: Distribution ratio of C methylated of different sequence types; **b, e**: Methylation patterns of methylation; **c, f**: Methylation patterns of different functional element regions in the whole genome.
(TIF)

S3 Fig Methylation levels of each chromosome for female tiger pufferfish (a) and pseudo male tiger pufferfish (b). (TIF)

S4 Fig Single base resolution methylation map for pseudo male (a, b), female (c, d) and male (e, f). From left to right was the starting point to the end point of chromosomes. The vertical axis represents 10 kb for calculation of the mC window density, with blue dots represent the distribution density of mC on the chromosomes, the vertical axis represents the standard ratio of mC, said the smooth curves of different types of methylation of C bases (CG, CHG and CHH) density distribution. The black part of the cross axle indicates the centromere.
(ZIP)
