# Supplementary figures and images for: Changes in DNA methylation during epigenetic-associated sex reversal under low temperature in Takifugu rubripes
Source: PLoS One. 2019 Aug 27;14(8):e0221641. doi: 10.1371/journal.pone.0221641 (PMC6711519; doi:10.1371/journal.pone.0221641)

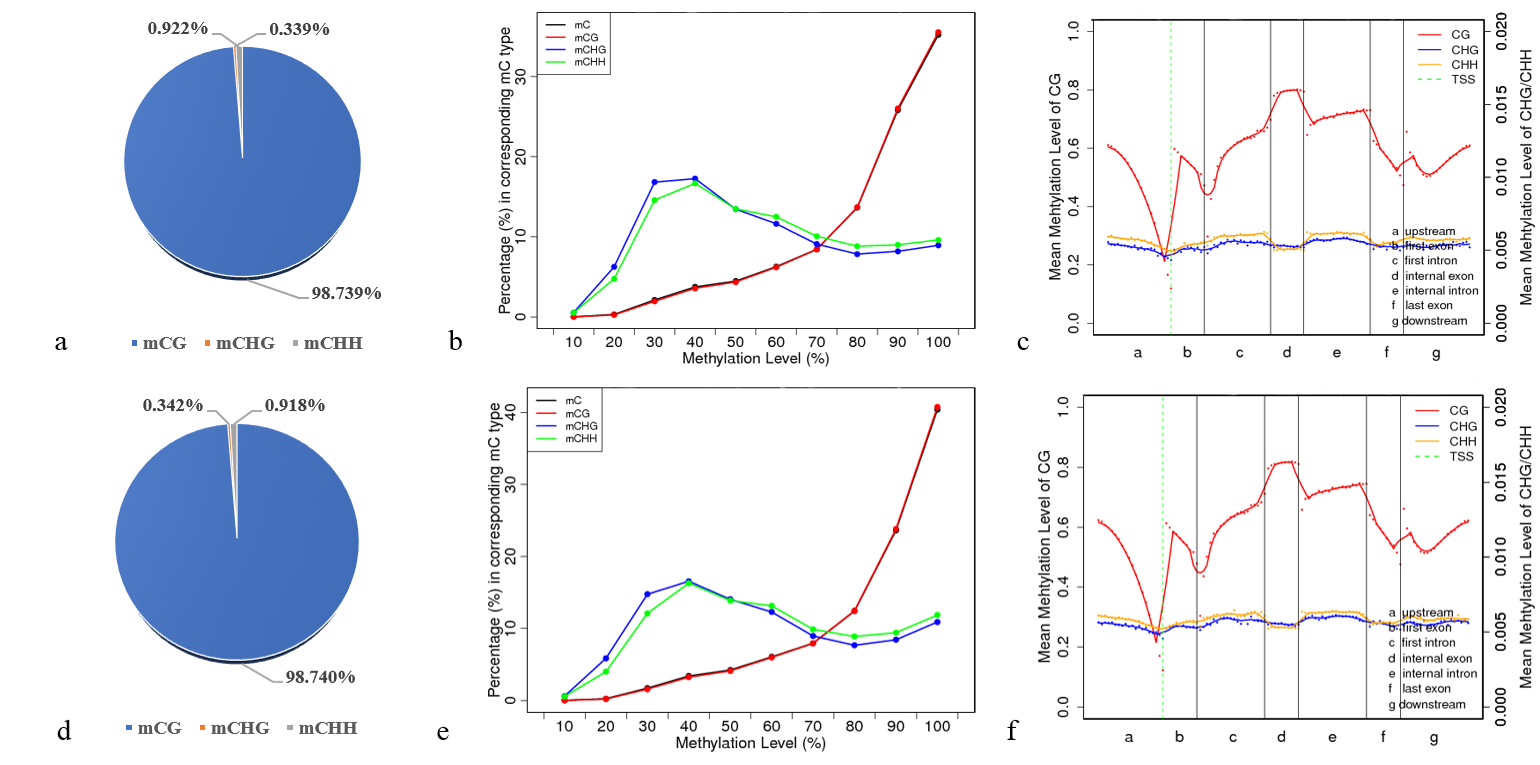

Supplement: S2 Fig — a, d: Distribution ratio of C methylated of different sequence types; b, e: Methylation patterns of methylation; c, f: Methylation patterns of different functional element regions in the whole genome. (TIF) [file pone.0221641.s005.tif]

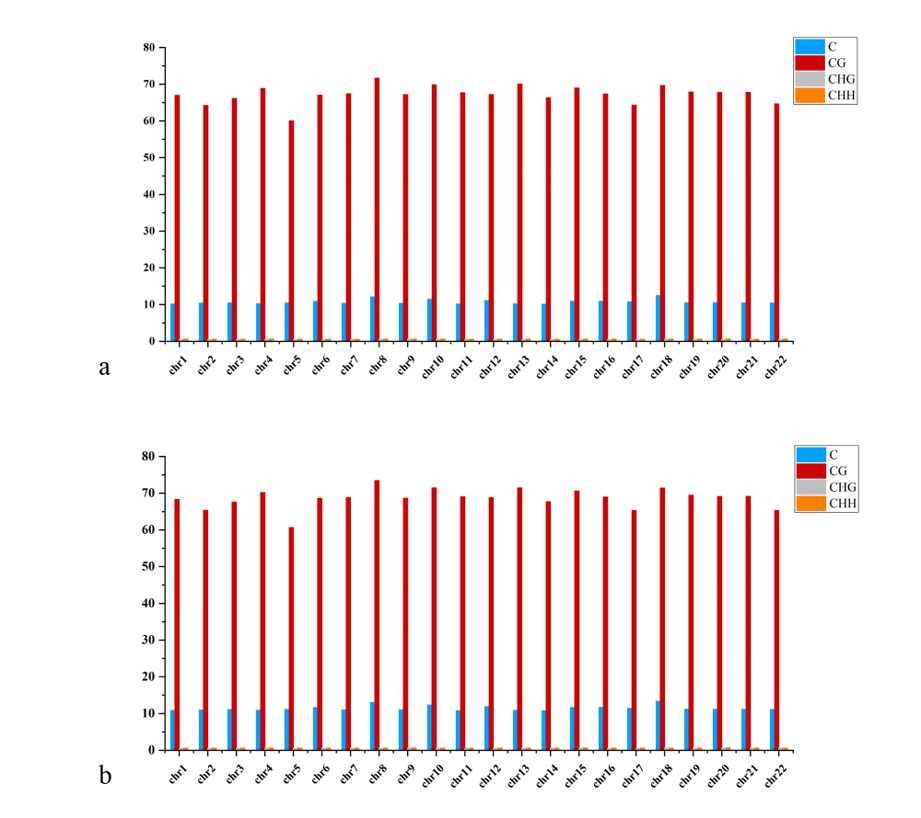

Supplement: S3 Fig — (TIF) [file pone.0221641.s006.tif]

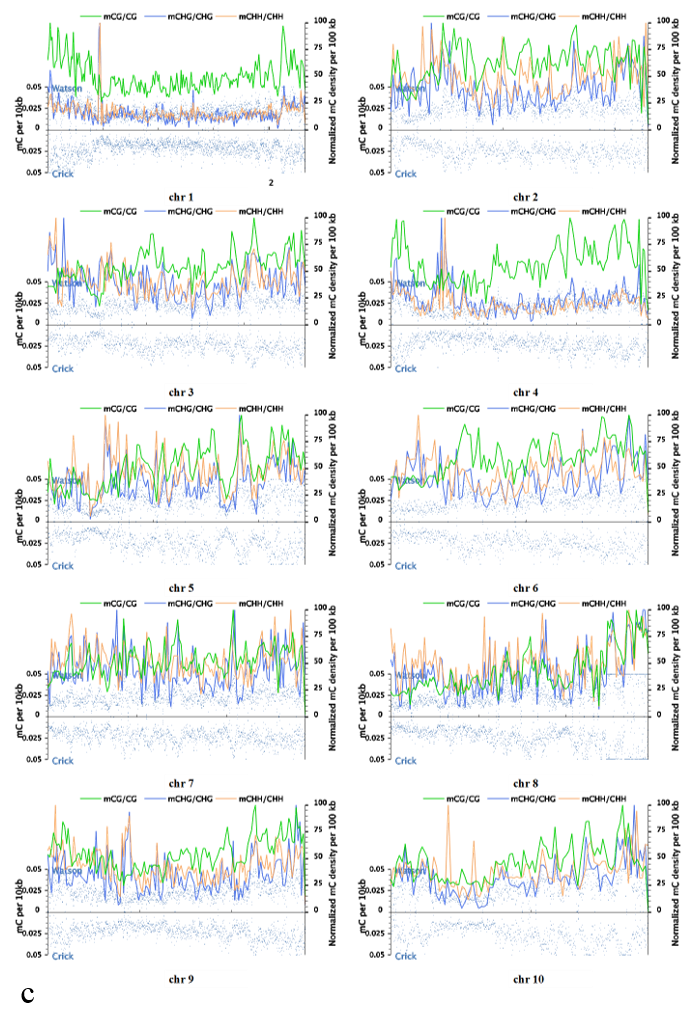

Supplement: S4 Fig — From left to right was the starting point to the end point of chromosomes. The vertical axis represents 10 kb for calculation of the mC window density, with blue dots represent the distribution density of mC on the chromosomes, the vertical axis represents the standard ratio of mC, said the smooth curves of different types of methylation of C bases (CG, CHG and CHH) density distribution. The black part of the cross axle indicates the centromere. (ZIP) [file pone.0221641.s007.zip › S4c_Fig .tif]

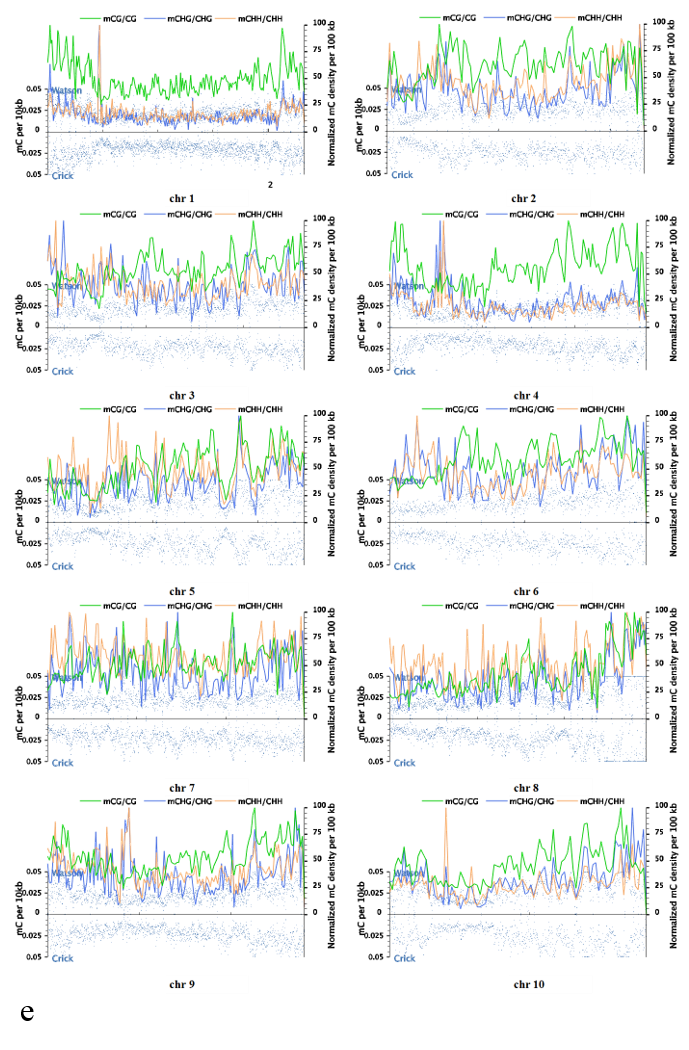

Supplement: S4 Fig — From left to right was the starting point to the end point of chromosomes. The vertical axis represents 10 kb for calculation of the mC window density, with blue dots represent the distribution density of mC on the chromosomes, the vertical axis represents the standard ratio of mC, said the smooth curves of different types of methylation of C bases (CG, CHG and CHH) density distribution. The black part of the cross axle indicates the centromere. (ZIP) [file pone.0221641.s007.zip › S4e_Fig.tif]

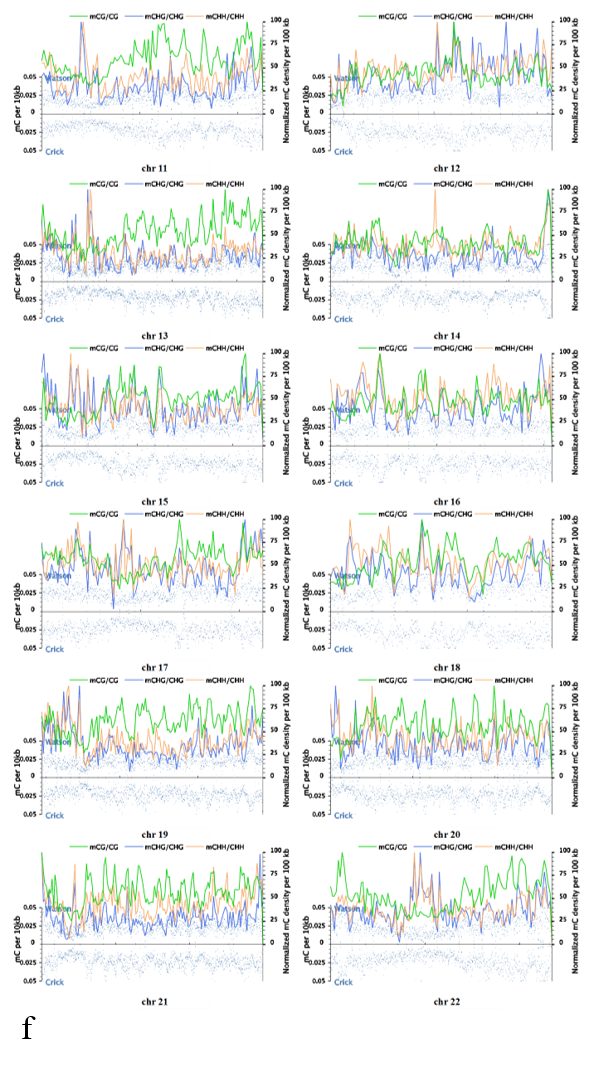

Supplement: S4 Fig — From left to right was the starting point to the end point of chromosomes. The vertical axis represents 10 kb for calculation of the mC window density, with blue dots represent the distribution density of mC on the chromosomes, the vertical axis represents the standard ratio of mC, said the smooth curves of different types of methylation of C bases (CG, CHG and CHH) density distribution. The black part of the cross axle indicates the centromere. (ZIP) [file pone.0221641.s007.zip › S4f_Fig.tif]

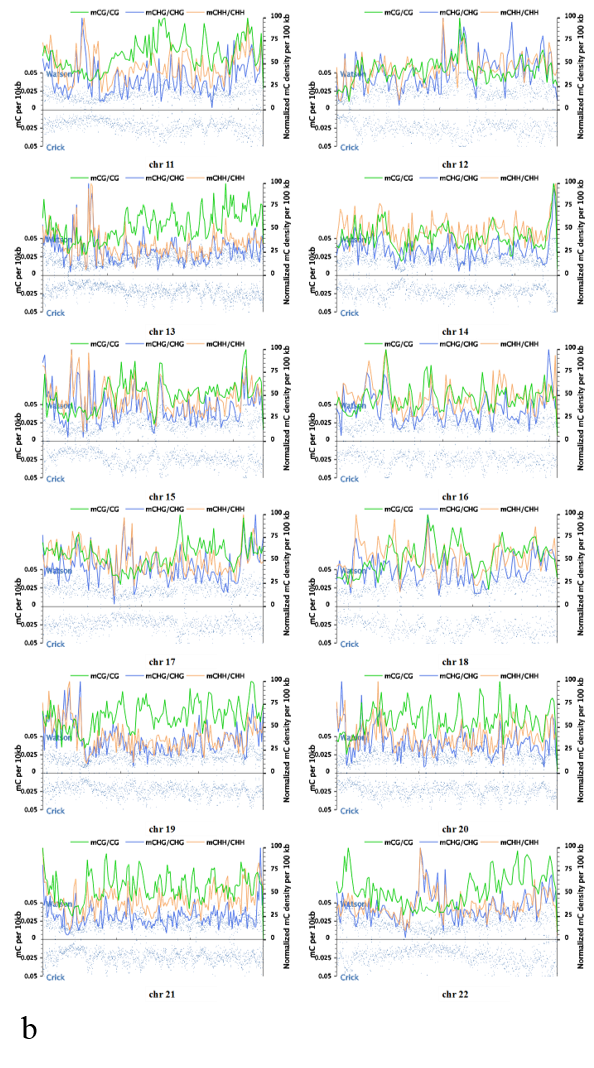

Supplement: S4 Fig — From left to right was the starting point to the end point of chromosomes. The vertical axis represents 10 kb for calculation of the mC window density, with blue dots represent the distribution density of mC on the chromosomes, the vertical axis represents the standard ratio of mC, said the smooth curves of different types of methylation of C bases (CG, CHG and CHH) density distribution. The black part of the cross axle indicates the centromere. (ZIP) [file pone.0221641.s007.zip › S4b_Fig .tif]

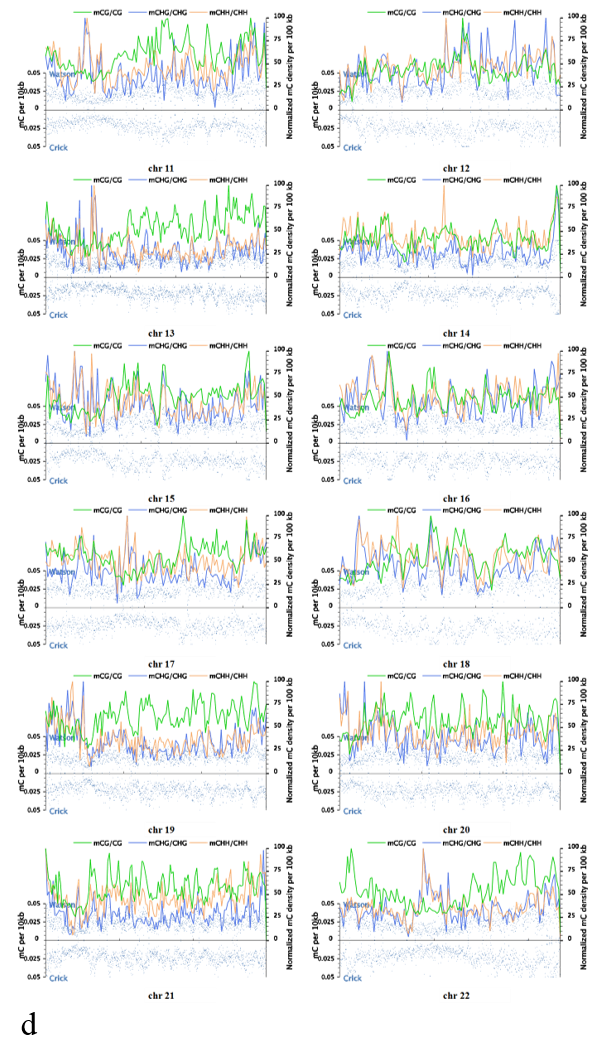

Supplement: S4 Fig — From left to right was the starting point to the end point of chromosomes. The vertical axis represents 10 kb for calculation of the mC window density, with blue dots represent the distribution density of mC on the chromosomes, the vertical axis represents the standard ratio of mC, said the smooth curves of different types of methylation of C bases (CG, CHG and CHH) density distribution. The black part of the cross axle indicates the centromere. (ZIP) [file pone.0221641.s007.zip › S4d_Fig.tif]

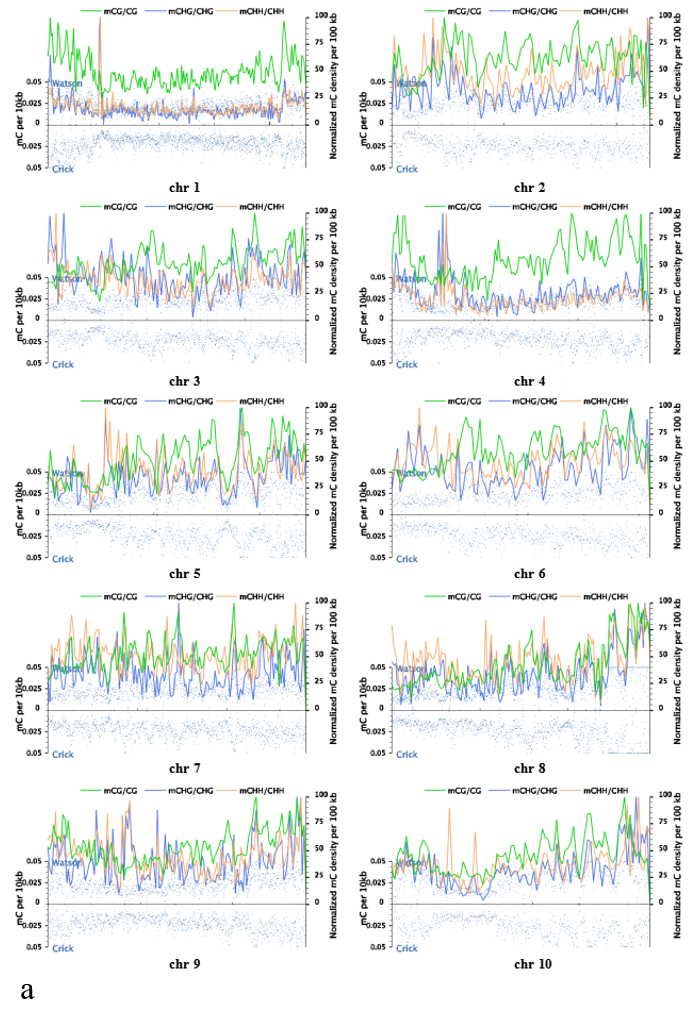

Supplement: S4 Fig — From left to right was the starting point to the end point of chromosomes. The vertical axis represents 10 kb for calculation of the mC window density, with blue dots represent the distribution density of mC on the chromosomes, the vertical axis represents the standard ratio of mC, said the smooth curves of different types of methylation of C bases (CG, CHG and CHH) density distribution. The black part of the cross axle indicates the centromere. (ZIP) [file pone.0221641.s007.zip › S4a_Fig.tif]
